# Physical Activity, Nutritional Habits, and Sleep Behavior Among Health Profession Students and Employees of a Swiss University During and After COVID-19 Confinement: Protocol for a Longitudinal Observational Study

**DOI:** 10.2196/25051

**Published:** 2020-12-22

**Authors:** Slavko Rogan, Eefje Luijckx, Jan Taeymans, Karin Haas, Heiner Baur

**Affiliations:** 1 Department of Health Professions Bern University of Applied Sciences Bern Switzerland

**Keywords:** healthy lifestyle, pandemic, public health, universities, COVID-19, SARS-CoV-2

## Abstract

**Background:**

SARS-CoV-2, a novel coronavirus strain, has resulted in the COVID-19 pandemic since early 2020. To contain the transmission of this virus, the Swiss Federal Council ordered a nationwide lockdown of all nonessential businesses. Accordingly, students and employees of institutions for higher education were informed to continue their academic programs through home-office settings and online lectures.

**Objective:**

This longitudinal survey aims to evaluate various lifestyle habits such as physical activity, nutritional habits, and sleep behavior among students and employees of a Swiss University of Applied Sciences during a 2-month period of confinement and social distancing due to the COVID-19 pandemic and 1 year thereafter.

**Methods:**

This paper describes a protocol for a retrospective and prospective observational cohort study. Students and employees of Bern University of Applied Sciences, Department of Health Professions, were invited to anonymously complete a web-based survey during the COVID-19 confinement period. This will be followed by a second survey, scheduled 1 year after the lockdown. Information on various lifestyle aspects, including physical activity, nutritional habits, and sleep behavior, will be collected using adaptations of existing validated questionnaires.

**Results:**

This longitudinal study started during the government-ordered confinement period in Switzerland in mid-April 2020 and will end in mid-2021.

**Conclusions:**

The findings of this survey will provide information about the impact of confinement during the COVID-19 crisis on the physical activity, nutritional habits, and sleep behavior of students and employees of a Swiss institute.

**Trial Registration:**

ClinicalTrials.gov NCT04502108; https://www.clinicaltrials.gov/ct2/show/NCT04502108

**International Registered Report Identifier (IRRID):**

DERR1-10.2196/25051

## Introduction

On March 11, 2020, the World Health Organization declared COVID-19 as a pandemic. Thereafter, on March 16, 2020, the Swiss Federal Council declared the pandemic as an extraordinary situation in accordance with the Epidemics Act from midnight of March 16, 2020, until April 19, 2020, during which stringent measures were introduced. All so-called “nonessential businesses” had to be closed, as well as schools of all levels, including universities and universities of applied sciences. The Swiss Federal Council called upon the members of the public to avoid all unnecessary social contact and to remain at home in order to maintain safe physical distance from other individuals, and as such, to contain the pandemic. This so-called extraordinary situation was extended further, and on May 11, 2020, most of the emergency packages were withdrawn.

These lockdown orders were immediately followed by the Head of the Bern University of Applied Sciences (BFH). Classroom teaching (ie, in lecture halls, classrooms, and skills rooms) was no longer possible, and students and employees were asked to remain at home and continue their work in home-office settings. During this period of confinement, social distancing, and working from home-office, lecturers were asked to switch to digital technologies to ensure continuation of various educational programs during the second half of the Spring 2020 academic semester and the upcoming Spring 2020/21 semester. The lifting of these strict COVID-19 measures is being closely monitored. The Swiss Federal Council can impose new restrictions in case of a sudden increase in new COVID-19 cases.

Lifestyle habits such as physical activity, nutritional habits, and sleep behavior of university lecturers and students during such an extraordinary period of confinement and social distancing have not been studied to date. However, there is ample evidence describing the positive effects of adequate physical activity as well as the negative effects of physical inactivity on human health [1-6]. During this nearly 2-month-long lockdown period, all sports infrastructure in Switzerland was forced to close. Although regular access to fitness clubs and sports facilities was no longer possible, individuals were allowed to continue walking, jogging, and cycling. Food shops, however, remained open during this confinement period, and Swiss residents were allowed to go outside for food supply while adhering to the recommended preventive measures (eg, maintaining safe physical distance). The positive relationship between healthy nutritional habits as well as good sleep behavior and improved human health has been well documented in the literature [7-9].

Because the COVID-19 pandemic is caused by a novel coronavirus strain, and vaccination and effective treatment options are currently lacking, it is difficult to predict how the pandemic will develop. Increased knowledge about lifestyle habits of students and employees of the Department of Health Professions (DHP) at BFH (BFH-DHP) during such an extreme confinement situation may help Heads and Deans of academic institutions to inform or counsel their students and employees during a similar situation, or in case of another outbreak, in the future. However, owing to the uniqueness of the ongoing COVID-19 crisis and its societal impact, such knowledge is currently lacking.

This longitudinal observational study will examine the impact of COVID-19 confinement on lifestyle habits such as physical activity, nutritional habits, and sleep behavior among students and employees of the BFH-DHP (Switzerland) during the 2-month confinement period and social distancing and 1 year after confinement.

## Methods

### Study Setting

**Figure 1:**
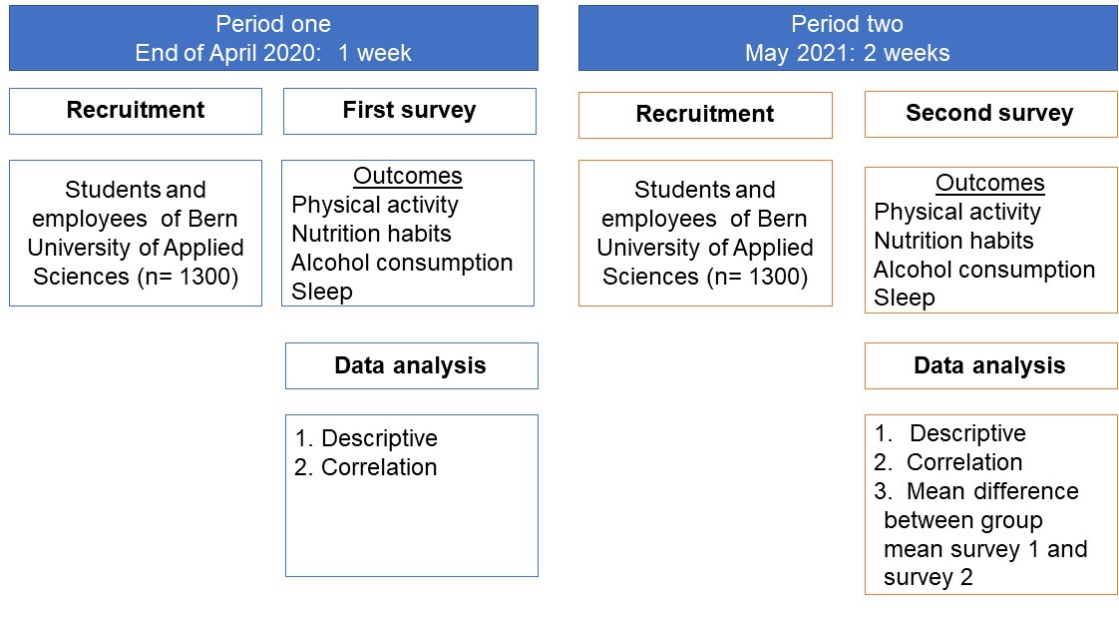
Flow diagram of the study.

This study follows a cohort study design and will be carried out at BFH-DHP in Switzerland. Figure 1 illustrates the flow diagram for the research design used in this study. This project is an interdisciplinary cooperation involving colleagues from the Departments of Nutrition and Dietetics and Physiotherapy of BFH-DHP. The contents of the survey will be developed at an interprofessional level.

This cohort study comprises 2 defined study periods: The first study period was in the end of April 2020, when the Swiss Federal Council declared the COVID-19 pandemic as an “extraordinary situation.” The first study was cross-sectional, consisting of an anonymous web-based survey. For the first survey, a questionnaire was sent to the staff and students of all 4 divisions of the institute during the COVID-19 confinement period. The participants’ responses were anonymous, in accordance with the data privacy policy of Switzerland [10]. Participants were not allowed to provide any personal information or other sociodemographic characteristics and living status. Participants could stop and exit the survey at any stage before completion. Anonymous survey procedures are known to yield higher disclosure rates of sensitive or stigmatizing information than nonanonymous procedures. Moreover, higher disclosure rates have traditionally known to be associated with more accurate results than lower disclosure rates [11].

The second survey will be conducted in May 2021; this survey will aim to collect information on the sociodemographic and living status of the participants. The time period for the first survey was 1 week and that for the second survey has been set to 2 weeks.

One researcher (SR) was responsible for the preparation of the electronic version of the first survey in April 2020 (during the full-confinement period); the same researcher will also complete implementation of the survey as a follow-up in May 2021. This study has been registered at ClinicalTrials.gov: NCT04502108.

### Ethical Approval and Consent to Participate

Prior to the planning of this protocol, the Dean of the Health Department of BFH was informed about the survey, and he approved this study. This cohort study will be performed following the ethical principles of the Declaration of Helsinki. In the introductory section of the survey, eligible participants (staff and students) were informed that the survey is voluntary and anonymous and that no medical data is requested. Participants could also contact the researchers for any information or further questions. Finally, participants were explicitly asked to provide informed consent for participation electronically, by clicking a button at the start of the electronic survey. The second survey will follow the same procedure outlined above. The evaluation system software we intend to use in this study does not allow for any tracing of the respondents.

The cantonal Ethics Committee of Bern is responsible for this research project. The main task of the ethics committees of Switzerland is to examine and approve applications for research projects in the field of human research. The committees evaluate projects that fall under the following definition from Article 2 of the Federal Act on Research involving Human Beings: “This Act applies to research concerning human diseases and concerning the structure and function of the human body, which involves: persons; deceased persons; embryos and foetuses; biological material; and health-related personal data.” We sought clarification from the Ethics Committee of Bern on whether this research project falls within the scope of the Human Research Act and/or to request a written statement in case the project does not need approval by the committee. We thus verified that this study does not need an approval from the ethics committee (Reference number: Kantonale Ethikkomission Bern, KEK Bern, Req-2020-00909).

### Recruitment

This cohort study aims to recruit at least 80% of the 1300 students and employees of BFH-DHP. The recruitment strategy comprised the following procedure: in consultation with and support of the director of BFH-DHP, an invitational letter will be sent via email to all students and employees during the first (April 2020) and second (May 2021) study periods.

### Eligibility Criteria

All students (N=1300; 88% female and 12% male) enrolled in programs at the Bachelor of Science and Master of Science levels as well as all academic and nonacademic employees of BFH-DHP are eligible and will be invited to participate in this comprehensive, web-based survey. BFH-DHP runs these educational programs in the fields of nursing, nutrition and dietetics, midwifery, and physiotherapy. Individuals that are not employed or enrolled at the university will not be eligible for this study.

### Outcomes

#### Primary Outcome (Physical Activity)

The validated German version of the international physical activity questionnaire-short form (iPAQ-SF) [12] will be used to assess the participants’ physical activity over the last 7 days. Seven items of the questionnaire will evaluate the number of days participants performed intensive and moderate physical activities and walking activities and the time (hours and minutes) spent per day in performing the exercises at those intensities. The primary outcome variable will be total physical activity expressed as metabolic equivalents of task in minutes per week (METs/week), which is calculated as the sum of 3 physical activities (ie, walking activity and moderate and intensive physical activities). For the categorical division into the 3 activity levels (low, moderate, and high), definitions from published evaluation guidelines will be used.

#### Secondary Outcomes

##### Nutritional Habits

The valid German version of the Mediterranean diet screener (bMDSC) [13] will be used to assess the participants’ nutritional habits and adherence to the Mediterranean diet. Participants will be asked to report their adherence to the recommended consumption frequency of 15 selected food items during the past year: (1) use of olive oil for cooking; (2) use of olive oil as salad dressing; (3) daily intake of cooked vegetables; (4) daily intake of raw vegetables; (5) daily intake of fruits; (6) daily intake of red meat or sausages; (7) intake of butter and bread for breakfast or dinner; (8) intake of soft drinks or sweetened drinks on the previous day; (9) attention to nutritional fiber intake; (10) weekly intake of legumes, chickpeas, and beans; (11) intake of fish 1-2 times per week; (12) consumption of nuts at least at 3 days per week; (13) preference of white meat over read meat; (14) intake of rice or pasta with vegetable sauce as part of the diet; and (15) consumption of French fries on the previous day [14]. Response categories will be “Yes” or “No.” All questions will be scored “1” if answered with “Yes,” except questions (6), (7), (8), and (15) that will be scored “1” if answered as “No.” Scores will be summed, and a higher score will indicate better adherence to the Mediterranean diet.

##### Alcohol Intake

Daily consumption of wine, beer, and spirits (liquor) will be evaluated and measured in units (glasses). For instance, how many units (glasses) of wine, beer, and spirits are consumed per day?

##### Sleep

Sleep behavior will be evaluated and measured as number of hours of sleep and quality of sleep via the following question: How well do you sleep? Possible answers include “No sleep problems,” “Sleep quality could be improved,” and “important sleep problems.”

#### Assessment Methods

##### First Survey

The questionnaire was sent via the institute’s email system to all eligible staff and students during the 2020 COVID-19 confinement period. A brief introduction section prefaced the questionnaires to explain the survey objectives. The survey was open during a brief, 1-week period to assure a full-confinement snapshot. Automated reminders were sent at least 2 times during this period. Submitted survey responses have been stored on the institute’s “evaluation system software,” from where data can be merged and extracted in an Excel spreadsheet for further analyses. As this data management will be fully automated, no special quality control procedures other than regular plausibility control measures (eg, range checks) are planned.

##### Second Survey

The same questionnaire that was used in the first survey will be sent to all eligible staff and students of BFH-DHP about 1 year later (ie, Spring 2021) during the period of “new normality.” The survey will be open during a 2-week period, and automated reminders will be sent to all participants. Because the process will be completely anonymous, the question “Did you volunteer to participate in the same survey last year?” will be added to the questionnaire. This question will allow to extract a set of participants that filled out both surveys and further perform group comparison analyses of lifestyle habits during confinement and under conditions of “new normality.” Furthermore, the second survey will include additional questions on gross anthropometric characteristics (ie, height and weight), sociodemographic characteristics, living status, gender, age, household size, changes in smoking habits, and quality of life.

#### Data Collection

This electronic survey will be conducted anonymously using the “evaluation system software” of BFH-DHP (EvaSys, Electric Paper EvaluationssystemeGmbH), which is typically used for quality control of lectures, seminars, and other educational products. The validated iPAQ-SF and bMDSC questionnaires will be transferred within the EvaSys-framework to assess participants’ physical activity and nutritional habits. Additional questions on alcohol consumption and sleep behavior will also be added to the survey.

The first survey will be conducted electronically. After closing of the 1-week survey period by the end of April 2020, the data collected will be merged and automatically extracted into an Excel spreadsheet for further analyses. The first survey will be analyzed as a cross-sectional study, in accordance with the official guidelines to analyze iPAQ-SF and adapted bMDSC. The survey results will be subjected to analyses as described in the Statistical Analyses section.

The second survey in May 2021 will also be analyzed as a cross-sectional study using the same procedures as used for the analysis of the first survey in 2020. In addition, a subgroup analyses of participants who volunteered in both surveys will allow for group comparison (during confinement versus after confinement).

#### Data Management

Data management will be performed on the BFH-DHP server. The EvaSys system will automatically store data collected from all study participants as comma separated variables (.csv) files. All data files will be imported and merged to a single data file in Excel (2018; Microsoft Corp).

Data cleansing will be performed by one researcher (JT) who will check and solve for example dot-comma decimal signs incompatibilities and to control plausibility of the data (ie, range checks). The iPAQ-SF data-cleansing guidelines will strictly be adhered to, that is, participants with incomplete (missing) data or those who responded “don’t know” will be removed from the analyses.

Data analyses will be carried out in 2 steps. First, data on physical activity, nutritional habits, alcohol consumption, and sleeping habits will be separately analyzed. Second, an explorative correlational analysis will be conducted, including only those participants with a complete set of data for all lifestyle habits under evaluation. Subgroup analyses will be conducted to compare differences in the outcomes between health profession divisions or between students’ academic levels (ie, BSc and MSc) and employee status.

#### Statistical Analyses

Parametric and nonparametric statistics will be used to report results from both surveys and to compare survey results with reference values obtained from the general population during pre–COVID-19 conditions. For descriptive analyses, central tendencies will be expressed as means or medians, and variation will be expressed as SD and 95% CI or interquartile ranges (25th and 75th percentiles), respectively. Kruskal-Wallis tests with posthoc Bonferroni corrections will be used to assess differences between independent groups. Results will be presented as frequency tables or as figures with boxplots.

For the explorative correlation analysis, health profession divisions, students’ academic level, and employee status will be recoded as follows: nutrition and dietetics = 1, midwifery = 2, nursing = 3, physiotherapy = 4, BSc = 1, MSc = 2, and employee = 3. The Spearman rank order correlation coefficient will be used to assess associations between the different variables under analysis. Results will be presented in a correlation matrix.

Statistical analyses will be conducted using SPSS software (version 26.0; IBM Corp.). Statistical significance will be set at the 5% level of error.

## Results

This longitudinal study was started during the lockdown in Switzerland, in mid-April 2020 and will be completed in May 2021. The first phase of the study is underway, and data from 823 participants will be evaluated.

## Discussion

This paper outlines the study protocol of the survey conducted among BFH-DHP students and employees to evaluate their physical activity, nutritional habits, and sleep behavior during the 2020 COVID-19 confinement. This protocol has been developed as an interdisciplinary collaboration between faculty members of the Divisions of Nutrition and Dietetics and Physiotherapy.

During the same period, similar initiatives were undertaken by other institutes. For example, a large survey developed by a consortium of many international universities aimed to retrospectively evaluate the changes in lifestyle habits among the general population in context of the COVID-19 pandemic [2,15,16].

One of the strengths of our survey is that it will be short (time needed to complete the survey will be less than 10 minutes), limiting the questions to physical activity, nutritional habits, and sleep behavior within a specific setting of students and employees of BFH-DHP. We believe, this will help the participants place special focus on the current and future multipliers of healthy lifestyle habits and their own personal behavioral changes due to a general lockdown effected as a key countermeasure to combat the spread of the COVID-19 pandemic. Another strength of our survey is its prospective design over a 1-year period. Based on the large sample size comprising eligible staff and students (N>1500) and considering the huge impact of COVID-19–related strict measures on both the staff and students, we expect a reasonable response rate (ie, around 30%). It may be hypothesized that such a confinement period may negatively affect physical activity levels of individuals, in general, and students and employees of BFH-DHP, in particular. In an effort to support the public in maintaining “healthy” physical activity levels, Swiss television channels resumed broadcasting home exercise programs after almost two decades. During this time, popular press reported an increase in the demand for fresh vegetables and fruit, as well as alcoholic beverages. Our survey will aim to verify and, if applicable, quantify such observations and put them in proper context.

The results of this survey may have important public health impacts. They may empower heads of universities and higher education institutions to better prepare, inform, and counsel their students and employees on maintaining healthy lifestyle habits that may prove useful, for example, in case of an upcoming second wave of COVID-19 or a sudden outbreak of a serious new viral epidemic or pandemic in the future. Action plans for workplace health promotions may be developed with a special focus on digital dissemination routes to reach students and employees in their home-office settings.

Several biases can occur in cohort studies, which should be reduced. For instance, a selection bias resulting from the approach used to select or observe study participants, which may influence the relationship between exposure and outcome. To avoid this type of bias, this study will recruit persons from only a single institution, ie, BFH-DHP. Second, an information bias could originate either from the individuals being observed, observers, or instruments used to evaluate the results. In prospective cohorts, information bias can usually be easily avoided since measures can be taken during scheduling by including all variables in the registration forms (instruments) so that no variables of interest are overlooked.

Nevertheless, this study has limitations. The survey does not collect any data about illnesses among participants during the first phase, which could likely influence a change in the targeted behaviors.

## Abbreviations

BFH: Bern University of Applied Sciences
bMDSC: brief Mediterranean diet screener
DHP: Department of Health Professions
iPAQ-SF: International Physical Activity Questionnaire-short form
